# Measuring equity in utilization of emergency obstetric care at Wolisso Hospital in Oromiya, Ethiopia: a cross sectional study

**DOI:** 10.1186/1475-9276-12-27

**Published:** 2013-04-22

**Authors:** Calistus Wilunda, Giovanni Putoto, Fabio Manenti, Maria Castiglioni, Gaetano Azzimonti, Wagari Edessa, Andrea Atzori, Mario Merialdi, Ana Pilar Betrán, Joshua Vogel, Bart Criel

**Affiliations:** 1 Doctors with Africa CUAMM, via san Francesco 126, Padua 35121, Italy; 2 Department of Statistical Sciences, University of Padua, via Cesare Battisti 241, Padua 35121, Italy; 3 Wolisso Hospital, P.O. Box 250, Wolisso, Ethiopia; 4 Zonal Health Department, South West Showa Zone, P.O. Box 253, Wolisso, Ethiopia; 5 Department of Reproductive Health and Research, World Health Organization, Geneva 27 1211, Switzerland; 6 University of Western Australia, 35 Stirling Highway Crawley, Perth, Australia; 7 Institute of Tropical Medicine, Nationalestraat 155, Antwerp 2000, Belgium

**Keywords:** Socio-economic status, Emergency obstetrics care, Maternal health, Access

## Abstract

**Introduction:**

Improving equity in access to services for the treatment of complications that arise during pregnancy and childbirth, namely Emergency Obstetric Care (EmOC), is fundamental if maternal and neonatal mortality are to be reduced. Consequently, there is a growing need to monitor equity in access to EmOC. The objective of this study was to develop a simple questionnaire to measure equity in utilization of EmOC at Wolisso Hospital, Ethiopia and compare the wealth status of EmOC users with women in the general population.

**Methods:**

Women in the Ethiopia 2005 Demographic and Health Survey (DHS) constituted our reference population. We cross-tabulated DHS wealth variables against wealth quintiles. Five variables that differentiated well across quintiles were selected to create a questionnaire that was administered to women at discharge from the maternity from January to August 2010. This was used to identify inequities in utilization of EmOC by comparison with the reference population.

**Results:**

760 women were surveyed. An *a posteriori* comparison of these 2010 data to the 2011 DHS dataset, indicated that women using EmOC were wealthier and more likely to be urban dwellers. On a scale from 0 (poorest) to 15 (wealthiest), 31% of women in the 2011 DHS sample scored less than 1 compared with 0.7% in the study population. 70% of women accessing EmOC belonged to the richest quintile with only 4% belonging to the poorest two quintiles. Transportation costs seem to play an important role.

**Conclusions:**

We found inequity in utilization of EmOC in favour of the wealthiest. Assessing and monitoring equitable utilization of maternity services is feasible using this simple tool.

## Introduction

Health equity is one of the most important outcomes of the performance of health systems [1]. Equity can be defined as ‘the absence of unfair and avoidable or remediable differences in health among population groups defined socially, economically, demographically or geographically’ [2]. Inequity in access to health care is also a critical barrier to achieving Millennium Development Goals 4 (reduce child mortality) and 5 (improve maternal health). Unless performance indicators are examined relative to the socioeconomic status of the population, an improvement in average statistics may hide persistent or worsening inequities in a society [3]. This is because health services generally tend to serve the wealthier populations more than those that are poorer [4,5]. As a result, measuring the utilization of services by poorer individuals has become a crucial task for policymakers and public health managers at all levels to assess equitable impact of interventions [6,7].

Equity in access to health services is defined as use of health services matching differential health needs of different social groups within the population [2]. When considering inequity, reproductive health stands out as the “worst of the worst” [8]. Indeed, in low-income countries, huge disparities exist between poorer and richer women regarding access to antenatal care, skilled birth attendance and cesarean section [8-11]. Those disparities are reflected in the stark imbalances in maternal and neonatal mortality across socio-economic strata, with the highest burden being experienced by the poorest groups [12,13]. Improving equity in access to services for the treatment of complications that arise during pregnancy and childbirth, namely Emergency Obstetric Care (EmOC), is therefore fundamental if maternal and neonatal mortality are to be reduced. Consequently, there is a growing need to monitor equity in access to EmOC. Although routine data collection through health information systems (HIS) would be ideal [11,14], data at national levels are more commonly collected by surveillance systems and surveys that are of little use at the point of care. Equity in access to health care is a multidimensional phenomenon. At health facility level it is however practical to assess the degree of health equity via levels of health services utilization [2]. Ideally, a useful tool to assess inequities in utilization of health services at point of care should be easily manageable, understandable and interpretable with an acceptable level of statistical precision.

With 676 maternal deaths per 100,000 live births [15], Ethiopia has one of the highest maternal mortality ratios worldwide [16]. It is also among the 10 countries with the highest numbers of intrapartum-related neonatal deaths and intrapartum stillbirths [12]. Inequities in access to maternity services are significant. The caesarean section rate is 0.1% in the poorest quintile compared to 7.2% in the richest quintile [15]. Problems in accessing maternal health care are mostly due to the perception that it is not necessary, inability to pay for treatment, lack of transportation or long distances to health facilities and cultural barriers [15].

In response to the health situation in the country, in 2005 the Ethiopian Government launched the Ethiopia Health Sector Development Program (HSDP) III [17]. It contained a range of policies and strategies, such as fee exemption for pregnant women and young children, community health insurance and the expansion of the number of health posts (HPs) and health centers (HCs). In addition, the program included human resources strategies such as “task shifting” and the “flooding strategy” for the accelerated production of health extension workers (providing care at the community level) and health officers (HOs), who provide care mainly at the health center level. Despite all of these strategies, a mid-term review of the program in 2008 found that the reduction of maternal and neonatal mortality in Ethiopia was far from achieving the MDG schedule due to “serious gaps in the implementation” [18].

In this context, the objectives of this study were: 1) to develop a simple tool to measure equity in utilization of EmOC at Wolisso Hospital in Oromiya, Ethiopia; and 2) to measure equity in utilization of EmOC by applying the tool and comparing the socio-economic status of users of the hospital with women in the general population.

## Methods

### Study population and setting

The study population consisted of women utilizing EmOC at Wolisso Hospital. Wolisso Hospital is a referral, private, non-profit facility located in Wolisso town (central Ethiopia, 115 km south-west of Addis Ababa), which is the capital of the Southwest Shoa Zone (SWSZ) in the Oromiya region. SWSZ has a population of about 1.1 million inhabitants and is served by 81 health facilities (one hospital, ten HCs, 53 HPs and 17 private clinics). Wolisso Hospital is the only facility that provides comprehensive EmOC in the SWSZ.

To promote equity in access to Wolisso Hospital, the price of health services are highly subsidized. The Oromiya Regional Health Bureau provides financial support to the hospital within the framework of a public-private partnership [19]. The hospital is currently an implementer of the National Health Service Extension Program by supporting the activities of health extension workers in the region.

In conducting this study, we primarily followed the methodology proposed by Pitchforth *et al.* in 2007 to develop a proxy wealth index for EmOC in Bangladesh [11]. This consisted of selecting proxy wealth variables, scoring the variables, validating the scores, and comparing the wealth status of women in the study sample with that of women in the general population.

The study design has three components: 1) development of the proxy wealth index; 2) using the wealth index variables to construct the survey questionnaire for women attending Wolisso Hospital for EmOC; and 3) comparing women attending EmOC in Wolisso with the general population of the region.

### Development of the proxy wealth index in Ethiopia

For the development of the proxy wealth index, we used data from the Ethiopia 2005 Demographic and Health Survey (DHS). The DHS programme represents the largest worldwide effort to obtain demographic and health data from nationally representative household surveys in developing countries (http://www.measuredhs.com). As the DHS use standardized questionnaires and methods, they are often considered the best available source of data for many health and demographic indicators in developing countries. The surveys are usually conducted periodically every five years. As our study population was women in the region of Oromiya, we selected a dataset of women from the national DHS aged 15–49 years with a previous birth and who usually resided in Oromiya. This constituted the reference population against which we compared the women accessing the hospital.

All proxy wealth variables available in the DHS were cross tabulated against the wealth quintiles in the DHS dataset (See Additional file 1 for list of all variables). Five variables that differentiated well across all quintiles and showed proportional progression from the lowest to the highest quintile were selected for our proxy wealth index (Additional file 2). These variables were: roof material, ownership of a table, type of toilet facility, ownership of a radio and educational attainment (Table 1).

**Table 1:** Crude and weighted wealth scores of selected wealth variables

**Wealth variable**	**Response options**	**Crude score**	**Rescaled crude score**	**Assigned weight**	**Weighted score**
Main roof material	Cement/concrete	4	1.00	5	5.00
	Corrugated iron	3	0.67		3.35
	Wood planks/wood	2	0.33		1.65
	Thatch / leaf/reed/bamboo	1	0.00		0.00
Type of toilet facility	Flush to septic tank/sewer system/pit latrine	6	1.00	4	4.00
	Ventilated improved pit latrine (VIP)	5	0.80		3.20
	Pit latrine with slab	4	0.60		2.40
	Pit latrine without slab / open pit	3	0.40		1.60
	Composting toilet	2	0.20		0.80
	No facility / bush / field	1	0.00		0.00
Educational attainment	Higher	6	1.00	3	3.00
	Complete secondary	5	0.80		2.40
	Incomplete secondary	4	0.60		1.80
	Complete primary	3	0.40		1.20
	Incomplete primary	2	0.20		0.60
	No education	1	0.00		0.00
Household owns table	Yes	2	1.00	2	2.00
	No	1	0.00		0.00
Household has radio	Yes	2	1.00	1	1.00
	No	1	0.00		0.00

The second step involved assigning wealth scores. Two categories of wealth scores were generated: crude scores and weighted scores using our assigned weights. Crude scores were assigned to show the relative importance of response options within each variable in reflecting the wealth status. For instance, for educational attainment, no education was scored as 1, incomplete primary education as 2, and so forth. Crude scores were then rescaled to take values of between 0 and 1 (Table 1). This was done to ensure that the final score was not influenced by the number of response options a variable had.

Some variables may be more influential in differentiating between the poorest and the wealthiest quintiles. We introduced weighting to reflect this relative significance. These weights ranged from 1 to 5, for each variable based on factor loadings obtained from factor analysis of the five selected variables. Factor analysis is a data-reduction technique used to identify a small number of factors that explain most of the variance observed in a larger number of variables, whereas factor loadings show how much each variable correlates with each factor; higher loadings imply greater correlations [20]. These weights were then multiplied by the rescaled crude scores to obtain weighted scores for each variable response option (Table 1). The sum of these scores provided an overall weighted score for each individual. Scores could range from 0 to 15.

Lastly, we assessed the validity and reliability of the weighted scores of the five variables. Factor analysis was performed for all DHS wealth variables (including education) to generate factor scores, and the first factor was taken to represent the wealth status [21]. This formed the “gold standard” upon which the scores of the five variables were compared. Validity and reliability were assessed using correlation and kappa analysis respectively [22]. Because scores of the five variables were not normally distributed, Spearman’s correlation was used to assess the validity.

The questionnaire was developed on the basis of the 2005 DHS and the data for our study were collected in 2010. We however seized the opportunity of using updated DHS 2011 data that became available soon after our data collection. We re-checked the validity and reliability of the weighted scores of the 5 variables using this 2011 DHS data by following the same process as described above.

### Development of the survey questionnaire and data collection

In order to assess the socio-economic status of the women attending EmOC in Wolisso Hospital, we prepared a questionnaire to be administered to the women. The questionnaire needed to be simple, easy to administer and pose limited inconvenience to the women, hence the need for a limited set of variables. The five variables selected by the process explained above were used to construct the questionnaire which also included information on parity, place of residence: woreda (district) and kebele (village). Kebeles located in big and small towns or trading centers were classified as urban, and the rest were classified as rural. We also collected information on the means and cost of transportation to the hospital. The questionnaire is presented in Additional file 3.

A midwife working from 8.00 to 17.00 was responsible for administering the questionnaire to all women who were being discharged from the maternity ward during these hours and had given informed consent. A piloting of the questionnaire had shown that it was easy to use and well understood by the respondents. The midwife received a brief orientation to the questionnaire. Data collection took place from January 18 to August 11, 2010.

### Comparison with women in the general population

To assess who was utilizing EmOC, data of EmOC users from the questionnaire were compared with that of parous women in the Oromiya 2011 DHS dataset based on parity, area of residence and the five proxy wealth variables. Wealth variables would be equally distributed in the two groups if there were no inequity. Stata commands that account for the complex sampling design and weighting of DHS data were used. The reported p values are corrected for the study design [23]. Additionally, the assigned weights in Table 1 were used to develop weighted scores for the women accessing maternity services at Wolisso Hospital. In the 2005 DHS dataset, cutoff points of quintiles generated, based on weighted scores i.e. <0.60, 0.60-1.95, 1.20-3.15, 3.20-5.70 and >5.70 for quintiles 1, 2, 3, 4 and 5, respectively, were used to divide the women in our study dataset into five groups, thus grouping users of EmOC into the quintiles that they would have belonged to in the general population based on the 2005 DHS. All analyses, except the comparison with women in the general population; which was done using Stata version 11, were done using SPSS version 16.

### Assessing for selection bias

The response rate of EmOC users was calculated as the proportion of women who were discharged from the maternity ward during the study period and who were interviewed. To assess the extent to which this sub-population of interviewed women is representative or not of the overall population of maternity users at the hospital in the study period, characteristics of interviewed and non-interviewed women were compared on the basis of routine data notified in the hospital registers using their in-patient numbers. The two groups of women were compared for age, district of residence, length of hospital stay and mode of delivery.

## Results

During the study period (January-August 2010), 1508 women were discharged from the maternity ward out of whom 760 (50.4%) were interviewed. In 228 of the 760 women interviewed (30%), the inpatient number could not be matched with the inpatient number in the hospital database because of recording errors. We therefore have only data on the details of the maternity admission for 532 (70%) of the total population of interviewed women. In addition, one woman with missing data on wealth variables was excluded from analysis. The 532 women who had received delivery services and who were interviewed did not differ from those not interviewed (n = 748) in terms of age and district of residence. However, the interviewed women were more likely to have delivered by caesarean section or by assisted vaginal delivery (i.e. vacuum extraction or forceps delivery) and to have stayed longer at the hospital following delivery (Additional file 4).

Table 2 shows the characteristics of women utilizing EmOC at Wolisso Hospital, compared with women in the general population in the Ethiopia 2011 DHS. Women utilizing EmOC were more likely to have a lower parity than women in the DHS sample (p < 0.001). There was a significant difference in place of residence, with 54.5% of users of EmOC residing in urban areas *versus* only 13.3% of women in the DHS sample (p < 0.001). There were also significant differences in all of the proxy wealth variables between women using EmOC at the hospital and those in the DHS sample, suggesting that users of EmOC were of a higher socioeconomic status than women in the general population.

**Table 2:** A comparison of the characteristics of women accessing emergency obstetrics care (EmOC) and those in the Oromiya 2011 Demographic and Health Survey (DHS) dataset

**Characteristic**	**Dataset n (%)**		**P value†**
	Oromiya 2011 DHS (n =4065*)	**Users of EmOC (n = 758)**	
**General**			
Urban residence	540 (13.3)	413 (54.5)	<0.001
Parity			<0.001
1	616 (15.2)	336 (44.7)	
2	657 (16.2)	153 (20.4)	
3	557 (13.7)	96 (12.8)	
>3	2235 (55.0)	166 (22.1)	
**Proxy wealth variables**			
Roof material			< 0.001
Thatch/leaf/reed	2137 (52.6)	218 (28.8)	
Wood planks	46 (1.1)	154 (20.3)	
Corrugated iron	1859 (45.8)	372 (49.1)	
Cement/concrete	22 (0.5)	14 (1.8)	
Type of toilet			< 0.001
No facility/bush/field	1799 (44.3)	95 (12.5)	
Composting toilet	116 (2.9)	33 (4.4)	
Pit latrine without slab/open pit	1808 (44.5)	182 (24.0)	
Pit latrine with slab	230 (5.7 )	426 (56.2)	
Ventilated improved pit latrine	49 (1.2)	15 (2.0)	
Flush toilet	60 (1.5)	7 (0.9)	
Education attainment			<0.001
No education	2639 (64.9)	281 (37.1)	
Incomplete primary	1107 (27.2)	197 (26.0)	
Complete primary	111 (2.7)	43 (5.7)	
Incomplete secondary	85 (2.1)	134 (17.7)	
Complete secondary	23 (0.6)	32 (4.2)	
Higher	100 (2.5)	71 (9.4)	
Has a table	1581 (38.9)	652 (86.0)	<0.001
Has a radio	1852 (45.6)	623 (82.1)	<0.001

*weighted using individual women weights in the DHS dataset.

†design based p values from F tests.

DHS Oromiya n = 4062 for table and toilet.

EmOC users n = 751 for parity. Overall, two women with missing data were excluded.

### Wealth scores

**Figure 1:**
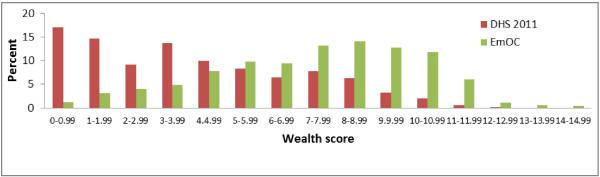
The distribution of wealth scores of women in the Oromiya 2011 Demographic and Health Survey sample and users of emergency obstetrics care (EmOC).

Figure 1 shows the distributions of the wealth scores of the 2011 DHS dataset and the study sample, using assigned weights of Table 1. Users of EmOC had higher wealth scores than did women in the general population. On a scale from 0 (poorest) to 15 (wealthiest), 17% of the women in the 2011 DHS sample had a score of less than 1 compared with less than 1% in the group of users of EmOC. Further analyses showed that the percentage of women who scored less than 5 in the 2011 DHS sample was 65% compared to 21% among users of EmOC. Only 3% of women in the DHS dataset scored 10 or more compared to 20% among EmOC users.

**Figure 2:**
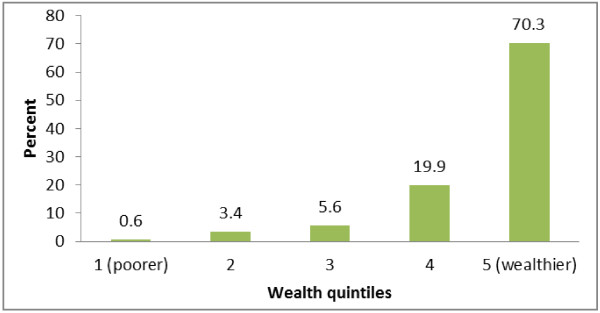
The distribution of women utilising emergency obstetrics care in wealth quintiles based on scores weighted with assigned weights.

Figure 2 shows the distributions of the women who utilized EmOC facility into quintiles based on weighted scores. About 70% of the women utilizing EmOC belonged to the richest 5^th^ quintile with only 4% of EmOC users coming from the two lowest quintiles combined.

There was a strong positive correlation between the “gold standard” and weighted scores of the five selected variables (correlation coefficient 0.876). When both scores were converted into quintiles, their correlation coefficient was 0.852. Validity of the questionnaire was thus established. The kappa statistic for weighted scores of the five selected variables and the “gold standard” was 0.464 (95% CI 0.435 - 0.493, p < 0.001), implying fair to good agreement. Revalidation of the tool using the 2011 DHS data still showed high validity (correlation coefficient 0.855), but a slightly reduced reliability (kappa 0.39, 95% CI 0.36-0.42).

### Transportation costs to the facility

Almost half of the women (47.4%) used public transportation to reach the hospital. 16% used a private car; 11.2% used bajaj, a small vehicle resembling a three-wheeled motorcycle; 10.8% went by foot; 10.3% in ambulance and 3.7% used a hand driven cart (3.7%). About 72% (546/760) of women had paid for transportation with a mean cost of 87 (SD 45) Birr.

## Discussion

In this study, we used the methodology proposed by Pitchforth et al. in 2007 [11] to develop a proxy wealth index. Applying this index, we showed a lower rate of utilization of EmOC at Wolisso Hospital by poorer women compared to wealthier women. Inequalities in utilization of EmOC by wealth quintile and residence are not new findings [24]. In the case of the utilization of EmOC services, social and financial barriers play a key role in many countries [25-27].

In our study, users of EmOC were of lower parity and more likely to be urban than rural dwellers. The rural–urban inequity is partly because the hospital is located in an urban setting. Given that it is the only hospital providing comprehensive EmOC in the region, rural women are disadvantaged by geography alone. The Oromiya region is crossed by one major road, and given that most women use public transportation to reach the facility, it is likely that women living further away from the main road had less access to it.

In an attempt to reduce barriers for poorer women, maternal health services provided by the hospital are largely subsidized for all women. For instance, the hospital charges 50 Birr for a normal delivery and 150 Birr for a caesarean section (1 Birr is about 0.06 US$). On the other hand, the mean cost of transport was 87 Birr with a cost for public transport of 55 Birr. Given that public transport cost was higher than the subsidized delivery fee, transportation expenditure is likely playing an important role in preventing poorer women from using the facility. Subsidizing transportation (for instance by using vouchers) is one potential mechanism to address this barrier.

The importance of non-financial barriers, such as concerns of quality of care or cultural issues, has also been described [28]. Wolisso Hospital is supported by a non-governmental organization (Doctors with Africa-CUAMM) and has built a good reputation in the region over the years. It began its services in January 2001, with the number of beds increasing from 83 in 2001 to 192 in 2010. Normal deliveries and cesarean sections also rose from 391 to 2,532 and from 54 to 493, respectively, over the same period. In 2010, the hospital’s bed occupancy rate was 90%. This steady growth of the hospital may indicate that quality and cultural issues are being addressed more effectively than financial barriers.

Financial barriers in accessing maternal care in Ethiopia are due to both direct and indirect costs. Indirect costs are partly due to long distances to health facilities. About 40% of the population in Ethiopia lives within 1 Km of any health facility [29]. Most women therefore have to pay for transportation costs; both for themselves and their companions, to access maternal care. Even though the Ethiopian government has tried to address the problem of direct costs by making maternal health services free of charge as stipulated in the HSDP III, most of the facilities that are supposed to be providing free services are only partially free in practice [18]. For instance, some women are charged for supplies used during delivery. With 39% of the population in Ethiopia living on less than 1.25 US$ a day [30], these costs still present a significant barrier to service access.

Importantly, the questionnaire was convenient and could be administered in less than 10 minutes. Weighted scores for each woman can be calculated and the woman categorized into a wealth group using cut-off points for weighted scores, without the need for statistical analysis. Because of these important features, the questionnaire can serve two functional roles. It can be used to identify poorer women for targeted social-assistance programs that take into account local determinants of poverty. Identification of the most deprived individuals is essential in implementing equity-focused interventions [31,32]. In relation to access to EmOC, attempts to better target poorer women for assistance has resulted in a number of innovative approaches, such as vouchers, equity funds and community health insurance [33,34]. Additionally, through periodic, low cost surveys, this questionnaire can be used to monitor the extent to which such programmes are contributing to the reduction of inequity in EmOC utilization.

Variables that capture the living standards are widely used in developing countries to measure the socio-economic status of households. In the Ethiopia 2005 DHS, almost 50 variables were used to construct the wealth index. In our study, we selected 5 variables that can be used to quickly assess the socio-economic status of service users in a hospital setting. One major challenge in limiting the number of variables to measure the wealth status is the issue of clumping and truncation [35]. Clumping is a situation whereby households are grouped into small number of distinct clusters while truncation is a situation where the socio-economic scores are spread over a narrow range. Both issues result into difficulties in differentiating between socio-economic groups. This is a typical problem in rural Ethiopia [36] where asset ownership and access to utilities is low and housing characteristics are the same for a majority of households. Pitchforth and colleagues [11] therefore suggest balancing the variables to include some whose possession in the poorer groups is average. Another solution is to increase the number of variables in the index [36], but this is not a good option if the goal is to a have an easy-to-construct and apply tool.

Our study has a number of limitations. The original plan to collect data from all women discharged from the hospital maternity in one full year was constrained mainly by the lack of human resources. During the study period (January – August 2010), the maternity ward sometimes ran out of beds and some women who had delivered without complications were discharged directly from the delivery ward without going through the postnatal ward. A good proportion of these women were thus missed. Of the 760 women who were interviewed, we could only trace back full data on maternity admission for 532 of them (i.e. 70%), because of problems in the routine hospital health information system. The women who were not interviewed (n = 748) did not differ from the interviewed sub-population (n = 532) based on age and district of residence. However, the two groups differed by mode of delivery and duration of hospital stay: the interviewed women being more likely to have a “complicated” delivery. Women with more severe maternal conditions are more likely to stay longer in the hospital and hence more likely to have been contacted for an interview irrespective of their socio-economic status. The effect of selection bias based on socio-economic status is thus thought to be minimal.

Although the administration of the questionnaire at discharge increased the convenience of data collection, it had the disadvantage of excluding the women who died and the runaway (i.e., women who left before being discharged). However, during the study period, hospital data showed six maternal deaths and no cases of runaway women. Seasonal variations in maternity service utilization may have affected our results, as the assessment did not cover a full year.

We revalidated the tool using the 2011 DHS data of parous women aged 15–49 years and usual residents of Oromiya. The validity of the tool had not changed much but its reliability had slightly reduced but still higher than what has been reported elsewhere [11]; implying that the tool may not have a stable reliability over time and hence the need to revalidate it using the most updated household survey data. One weakness of using variables that reflect living standards in the context of assessing access to health services is that these measures are reflective of cumulative household wealth and fail to take account of short-run or temporary interruptions to the households [37]. Moreover, the ownership of an asset does not reflect its quality or value. This has the potential of misclassifying individuals.

Ensuring access to EmOC to women of all socio-economic status is critical to improve maternal and newborn health and outcomes. Monitoring equity in service utilization at the point of care is indispensable given the current challenges facing poorer communities. Results from this study show that monitoring equity is feasible by applying a simple tool developed using the open-access DHS databases, internationally acknowledged and virtually ubiquitous in developing countries. The evidence of inequity that is highlighted here should prompt the implementation of effective strategies to promote equitable access to health services. Use of this tool to monitor the distribution of maternity service users by wealth quintiles is recommended. Further studies are needed to explain the contextual causes of unequal access to EmOC, in order to understand how best to address it.

## Ethical approval

The management and the ethical committee of Wolisso Hospital, Ethiopia.

## Competing interests

The authors declare that they have no competing interests.

## Authors’ contribution

GP and FM had the original idea and designed the study with CW. CW and MC conducted data analysis. CW wrote the first draft of the manuscript. GP, FM, MC, GA, WE, AA, APB, JV, MM and BC provided critical comments and valuable suggestions and contributed to writing of subsequent versions of the manuscript. All authors approved the final manuscript.

## Supplementary Material

Additional file 1 Proxy wealth variables in the Ethiopia DHS 2005. Click here for file

Additional file 2 Cross tabulation and selection of variables (only categorical variables presented). Click here for file

Additional file 3 Questionnaire developed and used in Wolisso Hospital. Click here for file

Additional file 4 A comparison of women interviewed and those not interviewed based on selected characteristics. Click here for file
