# Supplementary material for: Measuring equity in utilization of emergency obstetric care at Wolisso Hospital in Oromiya, Ethiopia: a cross sectional study
Source: Int J Equity Health. 2013 Apr 22;12:27. doi: 10.1186/1475-9276-12-27 (PMC3639914; doi:10.1186/1475-9276-12-27)
Supplement: Additional file 1 — Proxy wealth variables in the Ethiopia DHS 2005. [file 1475-9276-12-27-S1.doc]

**Measuring equity in utilization of emergency obstetric care at Wolisso Hospital in Oromiya, Ethiopia: a cross sectional study**

**Supplementary files - Annex 1**

**Proxy wealth variables in the Ethiopia DHS 2005**

Source of drinking water

Type of toilet facility

Has electricity

Has radio

Has television

Has refrigerator

Has bicycle

Has motorcycle/scooter

Has car/truck

Main floor material

Main wall material

Main roof material

Has telephone

Share toilet with other households

Type of cooking fuel

Disposal of household waste

Number of households sharing toilet

Household owns: watch

Household owns: mobile telephone

Household owns: table

Household owns: chair

Household owns: bed

Household owns: electric mitad

Household owns: kerosene / pressure lamp

Type of cooking fuel

Household has separate room used as kitchen

Main roof material

Main wall material

House has any windows

House has windows with glass

House has windows with screens

House has windows with curtains or shutters

Number of rooms used for sleeping

Animal-drawn cart

Boat without a motor

Boat with a motor

Land usable for agriculture

Hectares for agricultural land

Livestock, herds or farm animals

Cattle

Cows

Horses / donkeys / mules

Camels

Goats

Sheep

Chickens

Bank account

**Supplementary files - Annex 2**

**Cross tabulation and selection of variables (only categorical variables presented)**

|  | **Variables 1-5 were selected for inclusion in the proxy wealth index** | | | | | | |
| --- | --- | --- | --- | --- | --- | --- | --- |
|  | **Variable** | | **Wealth quintile (Column %)** | | | | |
|  |  |  | **1 Poorest** | **2** | **3** | **4** | **5** |
| 1 | Main roof material | |  |  |  |  |  |
|  |  | 12 Thatch / leaf | 97.3 | 87.1 | 79.1 | 46.7 | 3.0 |
|  |  | 22 Reed / bamboo | 2.2 | 3.8 | 1.3 | 0.7 | 0.0 |
|  |  | 23 Wood planks | 0.2 | 0.0 | 0.2 | 0.2 | 0.0 |
|  |  | 31 Corrugated iron | 0.2 | 8.1 | 19.2 | 52.0 | 96.0 |
|  |  | 32 Wood | 0.0 | 1.0 | 0.2 | 0.4 | 0.5 |
|  |  | 35 Cement / concrete | 0.0 | 0.0 | 0.0 | 0.0 | 0.5 |
| 2 | Type of toilet facility | |  |  |  |  |  |
|  |  | 11 Flush to piped sewer system | 0.0 | 0.0 | 0.0 | 0.0 | 1.6 |
|  |  | 12 Flush to septic tank | 0.0 | 0.0 | 0.0 | 0.0 | 1.1 |
|  |  | 13 Flush to pit latrine | 0.0 | 0.4 | 0.0 | 0.7 | 2.5 |
|  |  | 21 Ventilated improved pit latrine | 0.0 | 0.0 | 0.7 | 1.3 | 0.7 |
|  |  | 22 Pit latrine with slab | 0.0 | 0.0 | 0.0 | 2.0 | 25.4 |
|  |  | 23 Pit latrine without slab / open pit | 0.2 | 3.8 | 13.5 | 29.8 | 46.7 |
|  |  | 24 Composting toilet | 0.5 | 1.6 | 3.1 | 2.5 | 2.8 |
|  |  | 31 No facility / bush / field | 99.3 | 94.1 | 82.8 | 63.7 | 19.4 |
| 3 | Educational attainment | |  |  |  |  |  |
|  |  | 0 No education | 86.0 | 82.4 | 70.8 | 61.5 | 30.3 |
|  |  | 1 Incomplete primary | 13.2 | 16.6 | 25.7 | 30.0 | 25.5 |
|  |  | 2 Complete primary | 0.5 | 0.8 | 1.5 | 3.8 | 7.0 |
|  |  | 3 Incomplete secondary | 0.2 | 0.2 | 2.0 | 4.5 | 27.5 |
|  |  | 4 Complete secondary | 0.0 | 0.0 | 0.0 | 0.2 | 7.0 |
|  |  | 5 Higher | 0.0 | 0.0 | 0.0 | 0.0 | 2.6 |
| 4 | Household owns: table | |  |  |  |  |  |
|  |  | 0 No | 98.0 | 82.2 | 56.1 | 42.6 | 11.4 |
|  |  | 1 Yes | 2.0 | 17.8 | 43.9 | 57.4 | 88.6 |
| 5 | Has radio | |  |  |  |  |  |
|  |  | 0 No | 93.9 | 81.2 | 66.4 | 40.8 | 19.4 |
|  |  | 1 Yes | 6.1 | 18.8 | 33.6 | 59.2 | 80.6 |
| 7 | Has electricity | |  |  |  |  |  |
|  |  | 0 No | 100.0 | 100.0 | 100.0 | 99.6 | 34.0 |
|  |  | 1 Yes | 0.0 | 0.0 | 0.0 | 0.4 | 66.0 |
| 8 | Has television | |  |  |  |  |  |
|  |  | 0 No | 100.0 | 100.0 | 100.0 | 99.8 | 72.4 |
|  |  | 1 Yes | 0.0 | 0.0 | 0.0 | 0.2 | 27.6 |
| 9 | Has refrigerator | |  |  |  |  |  |
|  |  | 0 No | 100.0 | 100.0 | 99.1 | 99.3 | 88.3 |
|  |  | 1 Yes | 0.0 | 0.0 | 0.9 | 0.7 | 11.7 |
| 10 | Has bicycle | |  |  |  |  |  |
|  |  | 0 No | 99.8 | 99.8 | 99.8 | 100.0 | 89.8 |
|  |  | 1 Yes | 0.2 | 0.2 | 0.2 | 0.0 | 10.2 |
| 11 | Has motorcycle/scooter | |  |  |  |  |  |
|  |  | 0 No | 100.0 | 100.0 | 100.0 | 100.0 | 100.0 |
| 12 | Has car/truck | |  |  |  |  |  |
|  |  | 0 No | 100.0 | 100.0 | 100.0 | 100.0 | 98.4 |
|  |  | 1 Yes | 0.0 | 0.0 | 0.0 | 0.0 | 1.6 |
| 13 | Main floor material | |  |  |  |  |  |
|  |  | 11 Earth / sand | 85.8 | 78.6 | 74.3 | 71.4 | 38.9 |
|  |  | 12 Dung | 14.2 | 21.2 | 25.1 | 28.0 | 21.5 |
|  |  | 21 Wood planks | 0.0 | 0.0 | 0.7 | 0.0 | 1.1 |
|  |  | 22 Reed / bamboo | 0.0 | 0.0 | 0.0 | 0.7 | 4.2 |
|  |  | 31 Parquet or polished wood | 0.0 | 0.0 | 0.0 | 0.0 | 4.6 |
|  |  | 32 Vinyl | 0.0 | 0.0 | 0.0 | 0.0 | 5.5 |
|  |  | 33 Ceramic tiles | 0.0 | 0.0 | 0.0 | 0.0 | 0.4 |
|  |  | 34 Cement / bricks | 0.0 | 0.0 | 0.0 | 0.0 | 21.0 |
|  |  | 35 Carpet | 0.0 | 0.0 | 0.0 | 0.0 | 3.0 |
|  |  | 96 OTHER | 0.0 | 0.2 | 0.0 | 0.0 | 0.0 |
| 14 | Main wall material | |  |  |  |  |  |
|  |  | 11 No walls | 0.2 | 0.2 | 0.4 | 0.0 | 0.5 |
|  |  | 12 Cane / trunks / bamboo / reed | 7.4 | 4.2 | 0.0 | 1.1 | 0.2 |
|  |  | 21 Bamboo / wood wit | 90.4 | 92.7 | 98.0 | 96.9 | 85.0 |
|  |  | 22 Stone with mud | 0.2 | 0.8 | 1.1 | 0.9 | 1.8 |
|  |  | 31 Cement | 0.0 | 0.0 | 0.0 | 0.0 | 4.8 |
|  |  | 32 Stone with lime / cement | 0.0 | 0.0 | 0.0 | 0.0 | 4.8 |
|  |  | 33 Bricks | 0.0 | 0.0 | 0.0 | 0.0 | 1.1 |
|  |  | 34 Cement blocks | 0.0 | 0.0 | 0.0 | 0.0 | 1.6 |
|  |  | 36 Wood planks / shingles | 1.7 | 2.0 | 0.4 | 1.1 | 0.2 |
|  |  | 96 OTHER | 0.0 | 0.0 | 0.0 | 0.0 | 0.2 |
| 15 | Source of drinking water | |  |  |  |  |  |
|  |  | 11 Piped in dwelling | 0 | 0 | 0 | 0.2 | 1.1 |
|  |  | 12 Piped into compound | 0 | 0 | 0 |  | 26.9 |
|  |  | 13 Piped outside compound | 7.4 | 9.5 | 13.3 | 16.7 | 37.5 |
|  |  | 21 Unprotected well | 3.2 | 2.0 | 2.4 | 4.5 | 0.9 |
|  |  | 22 Unprotected spring | 2.2 | 3.2 | 8.3 | 7.1 | 5.6 |
|  |  | 31 Tube well or borehole | 0.0 | 0.0 | 0.2 | 0.0 | 0.0 |
|  |  | 32 Protected well | 1.2 | 4.8 | 0.9 | 2.2 | 1.6 |
|  |  | 33 Protected spring | 44.4 | 43.2 | 44.4 | 43.1 | 14.8 |
|  |  | 41River/dam/lake/pond/stream/canal | 41.2 | 37.2 | 30.3 | 26.1 | 11.6 |
|  |  | 51 Rainwater | 0.2 | 0.0 | 0.2 | 0.0 | 0.0 |
|  |  | 61 Tanker truck | 0.2 | 0.0 | 0.0 | 0.0 | 0.0 |
| 16 | Type of cooking fuel | |  |  |  |  |  |
|  |  | 1 Electricity | 0.0 | 0.0 | 0.0 | 0.0 | 0.9 |
|  |  | 2 LPG, natural gas | 0.0 | 0.0 | 0.0 | 0.0 | 0.2 |
|  |  | 4 Kerosene | 0.0 | 0.0 | 0.0 | 0.0 | 6.0 |
|  |  | 6 Charcoal | 0.0 | 0.0 | 0.0 | 0.0 | 9.5 |
|  |  | 7 Firewood, straw | 98.5 | 95.6 | 91.3 | 93.5 | 77.5 |
|  |  | 8 Dung | 1.5 | 4.2 | 8.7 | 6.5 | 5.8 |
|  |  | 96 Other | 0.0 | 0.2 | 0.0 | 0.0 | 0.2 |
| 17 | Disposal of household waste | |  |  |  |  |  |
|  |  | 1 Collected by municipality | 0.0 | 0.0 | 0.0 | 0.0 | 9.8 |
|  |  | 2 Collected by private establishment | 0.0 | 0.0 | 0.3 | 1.0 | 2.7 |
|  |  | 3 Dumped in street/open space | 99.2 | 93.6 | 91.8 | 82.8 | 38.4 |
|  |  | 4 Dumped in river | 0.0 | 0.5 | 0.5 | 0.3 | 5.7 |
|  |  | 5 Burned | 0.0 | 0.9 | 0.8 | 2.9 | 23.1 |
|  |  | 6 Other | 0.3 | 2.6 | 2.6 | 2.3 | 3.8 |
|  |  | 7 Dump inside hole | 0.5 | 2.4 | 4.1 | 8.4 | 16.4 |
|  |  | 8 Don't know | 0.0 | 0.0 | 0.0 | 2.3 | 0.0 |
| 18 | Household owns: watch | |  |  |  |  |  |
|  |  | 0 No | 80.6 | 60.6 | 43.6 | 28.9 | 15.7 |
|  |  | 1 Yes | 19.4 | 39.4 | 56.4 | 71.1 | 84.3 |
| 19 | Household owns: mobile telephone | |  |  |  |  |  |
|  |  | 0 No | 100.0 | 100.0 | 100.0 | 100.0 | 94.4 |
|  |  | 1 Yes | 0.0 | 0.0 | 0.0 | 0.0 | 5.6 |
| 20 | Household owns: chair | |  |  |  |  |  |
|  |  | 0 No | 87.0 | 76.0 | 56.6 | 41.7 | 17.1 |
|  |  | 1 Yes | 13.0 | 24.0 | 43.4 | 58.3 | 82.9 |
| 21 | Household owns: bed | |  |  |  |  |  |
|  |  | 0 No | 74.3 | 52.9 | 34.9 | 23.5 | 5.5 |
|  |  | 1 Yes | 25.7 | 47.1 | 65.1 | 76.5 | 94.5 |
| 22 | Household owns: electric mitad | |  |  |  |  |  |
|  |  | 0 No | 100.0 | 100.0 | 100.0 | 99.1 | 89.9 |
|  |  | 1 Yes | 0.0 | 0.0 | 0.0 | 0.9 | 10.1 |
| 23 | Household owns: kerosene / pressure lamp | |  |  |  |  |  |
|  |  | 0 No | 86.3 | 85.1 | 81.0 | 69.1 | 76.9 |
|  |  | 1 Yes | 13.7 | 14.9 | 19.0 | 30.9 | 23.1 |
| 24 | Type of cooking fuel | |  |  |  |  |  |
|  |  | 1 Electricity | 0.0 | 0.0 | 0.0 | 0.0 | 0.9 |
|  |  | 2 LPG | 0.0 | 0.0 | 0.0 | 0.0 | 0.2 |
|  |  | 5 Kerosene | 0.0 | 0.0 | 0.0 | 0.0 | 6.0 |
|  |  | 7 Charcoal | 0.0 | 0.0 | 0.0 | 0.0 | 9.5 |
|  |  | 8 Wood | 97.8 | 94.7 | 90.0 | 91.7 | 74.3 |
|  |  | 9 Straw / shrubs / grass | 0.7 | 0.8 | 1.3 | 1.8 | 3.2 |
|  |  | 11 Animal dung | 1.5 | 4.2 | 8.7 | 6.5 | 5.8 |
|  |  | 96 Other | 0.0 | 0.2 | 0.0 | 0.0 | 0.2 |
| 25 | Household has separate room used as kitchen | | |  |  |  |  |
|  |  | 0 No | 97.6 | 95.0 | 92.5 | 87.1 | 68.4 |
|  |  | 1 Yes | 2.4 | 5.0 | 7.5 | 12.9 | 31.6 |
| 26 | Animal-drawn cart | |  |  |  |  |  |
|  |  | 0 No | 100.0 | 99.6 | 99.6 | 99.3 | 97.0 |
|  |  | 1 Yes | 0.0 | 0.4 | 0.4 | 0.7 | 3.0 |
| 27 | Boat without a motor | |  |  |  |  |  |
|  |  | 0 No | 100.0 | 100.0 | 100.0 | 100.0 | 100.0 |
| 28 | Boat with a motor | |  |  |  |  |  |
|  |  | 0 No | 100.0 | 100.0 | 100.0 | 100.0 | 99.6 |
|  |  | 1 Yes | 0.0 | 0.0 | 0.0 | 0.0 | 0.4 |
| 29 | Land usable for agriculture | |  |  |  |  |  |
|  |  | 0 No | 3.7 | 9.1 | 7.2 | 9.4 | 62.1 |
|  |  | 1 Yes | 96.3 | 90.9 | 92.8 | 90.6 | 37.9 |

**Supplementary files - Annex 3**

Questionnaire developed and used in Wolisso Hospital

| **1** | Name of health facility | | | | |
| --- | --- | --- | --- | --- | --- |
| **2** | Questionnaire number |  | **3** | In-patient number |  |
| **4** | Today’s date (dd/mm/yy) | ___/____/___ | **5** | Admission date (dd/mm/yy) | ___/___/____ |
| **6** | Name of your Woreda | |  | | |
| **7** | Name of your Kebele | |  | | |
| **8** | Age in completed years | |  | | |
| **9** | Parity (*after the current delivery*) | |  | | |
| **10** | What main means of transportation did you use to come to this facility? | | 1. On foot 2. Bajaj 3. Private/hired car 4. Public transport 5. Other (specify)_________________________ | | |
| **11** | How much money did you spent on transportation to come here? | |  | | |
| **12** | Have you ever attended school? | | 1. No *15* 2. Yes | | |
| **13** | If yes, what is the highest level of school you attended? | | 1. Primary 2. Secondary 3. College/university *15* | | |
| **14** | What is the highest class you completed? | |  | | |
| **15** | Highest educational attainment  (*work out from 12, 13 & 14*) | | 1. No education 2. Incomplete primary 3. Complete primary 4. Incomplete secondary 5. Complete secondary 6. Higher | | |
| **16** | Do you have a table in your house? | | 1. No 2. Yes | | |
| **17** | Does your household own a radio? | | 1. No 2. Yes | | |
| **18** | What is the main material of the roof of your main house? | | 1. Thatch/leaf/reed/bamboo 2. Wood planks/wood 3. Corrugated iron 4. Cement/concrete 5. Other (specify)_______________________ | | |
| **19** | Do you have a toilet at home? | | 1. No *21*  2. Yes | | |
| **20** | What is the type of the toilet facility? | | 1. No facility / bush / field 2. Composting toilet 3. Pit latrine without slab / open pit 4. Pit latrine with slab 5. Ventilated improved pit latrine (VIP) 6. Flush toilet | | |
| **21** | Which service did the mother receive?  (*from the mother’s file*) | | 1. Normal delivery 2. Assisted vaginal delivery 3. Caesarean section 4. Other (specify)_________________________ | | |

**Supplementary files - Annex 4**

A comparison of between women interviewed and those not interviewed based on selected characteristics

| Characteristics | | N (%) | | Total | X^2^ test  P value |
| --- | --- | --- | --- | --- | --- |
|  |  | Not interviewed | Interviewed |  |  |
| District | Wolisso | 380 (50.8) | 272 (51.1) | 652 (50.9) | 0.789 |
|  | Goro | 42 (5.1) | 27 (5.1) | 69 (5.4) |  |
|  | Wonchi | 60 (8.0) | 34 (6.4) | 94 (7.3) |  |
|  | Welkite | 96 (12.8) | 73 (13.7) | 169 (13.2) |  |
|  | Becho | 44 (5.9) | 38 (7.1) | 82 (6.4) |  |
|  | Ameya | 30 (4.0) | 26 (4.9) | 56 (4.4) |  |
|  | Others | 96 (12.8) | 62 (11.7) | 158 (12.3) |  |
| Age | <20 | 82 (11.0) | 60 (11.3) | 142 (11.1) | 0.346 |
|  | 20-24 | 246 (32.9) | 195 (36.7) | 441 (34.5) |  |
|  | 25-29 | 256 (34.2) | 158 (29.7) | 414 (32.3) |  |
|  | 30-34 | 94 (12.6) | 71 (13.3) | 165 (12.9) |  |
|  | 35-39 | 52 (7.0) | 42 (7.9) | 94 (7.3) |  |
|  | 40-44 | 13 (1.7) | 5 (0.9) | 18 (1.4) |  |
|  | >44 | 5 (0.7) | 1 (0.2) | 6 (0.5) |  |
| Length of stay (days) | 0 | 100 (13.4) | 35 (6.6) | 135 (10.5) | <0.001 |
|  | 1 | 425 (56.8) | 239 (44.9) | 664 (51.9) |  |
|  | 2 | 58 (7.8) | 52 (9.8) | 110 (8.6) |  |
|  | 3 | 30 (4.0) | 24 (4.5) | 54 (4.2) |  |
|  | 4 | 20 (2.7) | 32 (6.0) | 52 (4.1) |  |
|  | 5 | 20 (2.7) | 29 (5.5) | 49 (3.8) |  |
|  | 6 | 19 (2.5) | 30 (5.6) | 49 (3.8) |  |
|  | >6 | 76 (10.2) | 91 (17.1) | 167 (13.0) |  |
| Type of delivery | Normal delivery | 668 (89.3) | 387 (72.7) | 1055 (82.4) | <0.001 |
|  | Caesarean section  or assisted vaginal* | 80 (10.7) | 145 (27.3) | 225 (17.6) |  |
| Total | | 748 (100) | 532 (100) | 1280 (100) |  |

*Forceps or vacuum extraction
