# Supplementary material for: Measuring equity in utilization of emergency obstetric care at Wolisso Hospital in Oromiya, Ethiopia: a cross sectional study
Source: Int J Equity Health. 2013 Apr 22;12:27. doi: 10.1186/1475-9276-12-27 (PMC3639914; doi:10.1186/1475-9276-12-27)
Supplement: Additional file 2 — Cross tabulation and selection of variables (only categorical variables presented). [file 1475-9276-12-27-S2.doc]

**Supplementary files - Annex 2**

**Cross tabulation and selection of variables (only categorical variables presented)**

|  | **Variables 1-5 were selected for inclusion in the proxy wealth index** | | | | | | |
| --- | --- | --- | --- | --- | --- | --- | --- |
|  | **Variable** | | **Wealth quintile (Column %)** | | | | |
|  |  |  | **1 Poorest** | **2** | **3** | **4** | **5** |
| 1 | Main roof material | |  |  |  |  |  |
|  |  | 12 Thatch / leaf | 97.3 | 87.1 | 79.1 | 46.7 | 3.0 |
|  |  | 22 Reed / bamboo | 2.2 | 3.8 | 1.3 | 0.7 | 0.0 |
|  |  | 23 Wood planks | 0.2 | 0.0 | 0.2 | 0.2 | 0.0 |
|  |  | 31 Corrugated iron | 0.2 | 8.1 | 19.2 | 52.0 | 96.0 |
|  |  | 32 Wood | 0.0 | 1.0 | 0.2 | 0.4 | 0.5 |
|  |  | 35 Cement / concrete | 0.0 | 0.0 | 0.0 | 0.0 | 0.5 |
| 2 | Type of toilet facility | |  |  |  |  |  |
|  |  | 11 Flush to piped sewer system | 0.0 | 0.0 | 0.0 | 0.0 | 1.6 |
|  |  | 12 Flush to septic tank | 0.0 | 0.0 | 0.0 | 0.0 | 1.1 |
|  |  | 13 Flush to pit latrine | 0.0 | 0.4 | 0.0 | 0.7 | 2.5 |
|  |  | 21 Ventilated improved pit latrine | 0.0 | 0.0 | 0.7 | 1.3 | 0.7 |
|  |  | 22 Pit latrine with slab | 0.0 | 0.0 | 0.0 | 2.0 | 25.4 |
|  |  | 23 Pit latrine without slab / open pit | 0.2 | 3.8 | 13.5 | 29.8 | 46.7 |
|  |  | 24 Composting toilet | 0.5 | 1.6 | 3.1 | 2.5 | 2.8 |
|  |  | 31 No facility / bush / field | 99.3 | 94.1 | 82.8 | 63.7 | 19.4 |
| 3 | Educational attainment | |  |  |  |  |  |
|  |  | 0 No education | 86.0 | 82.4 | 70.8 | 61.5 | 30.3 |
|  |  | 1 Incomplete primary | 13.2 | 16.6 | 25.7 | 30.0 | 25.5 |
|  |  | 2 Complete primary | 0.5 | 0.8 | 1.5 | 3.8 | 7.0 |
|  |  | 3 Incomplete secondary | 0.2 | 0.2 | 2.0 | 4.5 | 27.5 |
|  |  | 4 Complete secondary | 0.0 | 0.0 | 0.0 | 0.2 | 7.0 |
|  |  | 5 Higher | 0.0 | 0.0 | 0.0 | 0.0 | 2.6 |
| 4 | Household owns: table | |  |  |  |  |  |
|  |  | 0 No | 98.0 | 82.2 | 56.1 | 42.6 | 11.4 |
|  |  | 1 Yes | 2.0 | 17.8 | 43.9 | 57.4 | 88.6 |
| 5 | Has radio | |  |  |  |  |  |
|  |  | 0 No | 93.9 | 81.2 | 66.4 | 40.8 | 19.4 |
|  |  | 1 Yes | 6.1 | 18.8 | 33.6 | 59.2 | 80.6 |
| 7 | Has electricity | |  |  |  |  |  |
|  |  | 0 No | 100.0 | 100.0 | 100.0 | 99.6 | 34.0 |
|  |  | 1 Yes | 0.0 | 0.0 | 0.0 | 0.4 | 66.0 |
| 8 | Has television | |  |  |  |  |  |
|  |  | 0 No | 100.0 | 100.0 | 100.0 | 99.8 | 72.4 |
|  |  | 1 Yes | 0.0 | 0.0 | 0.0 | 0.2 | 27.6 |
| 9 | Has refrigerator | |  |  |  |  |  |
|  |  | 0 No | 100.0 | 100.0 | 99.1 | 99.3 | 88.3 |
|  |  | 1 Yes | 0.0 | 0.0 | 0.9 | 0.7 | 11.7 |
| 10 | Has bicycle | |  |  |  |  |  |
|  |  | 0 No | 99.8 | 99.8 | 99.8 | 100.0 | 89.8 |
|  |  | 1 Yes | 0.2 | 0.2 | 0.2 | 0.0 | 10.2 |
| 11 | Has motorcycle/scooter | |  |  |  |  |  |
|  |  | 0 No | 100.0 | 100.0 | 100.0 | 100.0 | 100.0 |
| 12 | Has car/truck | |  |  |  |  |  |
|  |  | 0 No | 100.0 | 100.0 | 100.0 | 100.0 | 98.4 |
|  |  | 1 Yes | 0.0 | 0.0 | 0.0 | 0.0 | 1.6 |
| 13 | Main floor material | |  |  |  |  |  |
|  |  | 11 Earth / sand | 85.8 | 78.6 | 74.3 | 71.4 | 38.9 |
|  |  | 12 Dung | 14.2 | 21.2 | 25.1 | 28.0 | 21.5 |
|  |  | 21 Wood planks | 0.0 | 0.0 | 0.7 | 0.0 | 1.1 |
|  |  | 22 Reed / bamboo | 0.0 | 0.0 | 0.0 | 0.7 | 4.2 |
|  |  | 31 Parquet or polished wood | 0.0 | 0.0 | 0.0 | 0.0 | 4.6 |
|  |  | 32 Vinyl | 0.0 | 0.0 | 0.0 | 0.0 | 5.5 |
|  |  | 33 Ceramic tiles | 0.0 | 0.0 | 0.0 | 0.0 | 0.4 |
|  |  | 34 Cement / bricks | 0.0 | 0.0 | 0.0 | 0.0 | 21.0 |
|  |  | 35 Carpet | 0.0 | 0.0 | 0.0 | 0.0 | 3.0 |
|  |  | 96 OTHER | 0.0 | 0.2 | 0.0 | 0.0 | 0.0 |
| 14 | Main wall material | |  |  |  |  |  |
|  |  | 11 No walls | 0.2 | 0.2 | 0.4 | 0.0 | 0.5 |
|  |  | 12 Cane / trunks / bamboo / reed | 7.4 | 4.2 | 0.0 | 1.1 | 0.2 |
|  |  | 21 Bamboo / wood wit | 90.4 | 92.7 | 98.0 | 96.9 | 85.0 |
|  |  | 22 Stone with mud | 0.2 | 0.8 | 1.1 | 0.9 | 1.8 |
|  |  | 31 Cement | 0.0 | 0.0 | 0.0 | 0.0 | 4.8 |
|  |  | 32 Stone with lime / cement | 0.0 | 0.0 | 0.0 | 0.0 | 4.8 |
|  |  | 33 Bricks | 0.0 | 0.0 | 0.0 | 0.0 | 1.1 |
|  |  | 34 Cement blocks | 0.0 | 0.0 | 0.0 | 0.0 | 1.6 |
|  |  | 36 Wood planks / shingles | 1.7 | 2.0 | 0.4 | 1.1 | 0.2 |
|  |  | 96 OTHER | 0.0 | 0.0 | 0.0 | 0.0 | 0.2 |
| 15 | Source of drinking water | |  |  |  |  |  |
|  |  | 11 Piped in dwelling | 0 | 0 | 0 | 0.2 | 1.1 |
|  |  | 12 Piped into compound | 0 | 0 | 0 |  | 26.9 |
|  |  | 13 Piped outside compound | 7.4 | 9.5 | 13.3 | 16.7 | 37.5 |
|  |  | 21 Unprotected well | 3.2 | 2.0 | 2.4 | 4.5 | 0.9 |
|  |  | 22 Unprotected spring | 2.2 | 3.2 | 8.3 | 7.1 | 5.6 |
|  |  | 31 Tube well or borehole | 0.0 | 0.0 | 0.2 | 0.0 | 0.0 |
|  |  | 32 Protected well | 1.2 | 4.8 | 0.9 | 2.2 | 1.6 |
|  |  | 33 Protected spring | 44.4 | 43.2 | 44.4 | 43.1 | 14.8 |
|  |  | 41River/dam/lake/pond/stream/canal | 41.2 | 37.2 | 30.3 | 26.1 | 11.6 |
|  |  | 51 Rainwater | 0.2 | 0.0 | 0.2 | 0.0 | 0.0 |
|  |  | 61 Tanker truck | 0.2 | 0.0 | 0.0 | 0.0 | 0.0 |
| 16 | Type of cooking fuel | |  |  |  |  |  |
|  |  | 1 Electricity | 0.0 | 0.0 | 0.0 | 0.0 | 0.9 |
|  |  | 2 LPG, natural gas | 0.0 | 0.0 | 0.0 | 0.0 | 0.2 |
|  |  | 4 Kerosene | 0.0 | 0.0 | 0.0 | 0.0 | 6.0 |
|  |  | 6 Charcoal | 0.0 | 0.0 | 0.0 | 0.0 | 9.5 |
|  |  | 7 Firewood, straw | 98.5 | 95.6 | 91.3 | 93.5 | 77.5 |
|  |  | 8 Dung | 1.5 | 4.2 | 8.7 | 6.5 | 5.8 |
|  |  | 96 Other | 0.0 | 0.2 | 0.0 | 0.0 | 0.2 |
| 17 | Disposal of household waste | |  |  |  |  |  |
|  |  | 1 Collected by municipality | 0.0 | 0.0 | 0.0 | 0.0 | 9.8 |
|  |  | 2 Collected by private establishment | 0.0 | 0.0 | 0.3 | 1.0 | 2.7 |
|  |  | 3 Dumped in street/open space | 99.2 | 93.6 | 91.8 | 82.8 | 38.4 |
|  |  | 4 Dumped in river | 0.0 | 0.5 | 0.5 | 0.3 | 5.7 |
|  |  | 5 Burned | 0.0 | 0.9 | 0.8 | 2.9 | 23.1 |
|  |  | 6 Other | 0.3 | 2.6 | 2.6 | 2.3 | 3.8 |
|  |  | 7 Dump inside hole | 0.5 | 2.4 | 4.1 | 8.4 | 16.4 |
|  |  | 8 Don't know | 0.0 | 0.0 | 0.0 | 2.3 | 0.0 |
| 18 | Household owns: watch | |  |  |  |  |  |
|  |  | 0 No | 80.6 | 60.6 | 43.6 | 28.9 | 15.7 |
|  |  | 1 Yes | 19.4 | 39.4 | 56.4 | 71.1 | 84.3 |
| 19 | Household owns: mobile telephone | |  |  |  |  |  |
|  |  | 0 No | 100.0 | 100.0 | 100.0 | 100.0 | 94.4 |
|  |  | 1 Yes | 0.0 | 0.0 | 0.0 | 0.0 | 5.6 |
| 20 | Household owns: chair | |  |  |  |  |  |
|  |  | 0 No | 87.0 | 76.0 | 56.6 | 41.7 | 17.1 |
|  |  | 1 Yes | 13.0 | 24.0 | 43.4 | 58.3 | 82.9 |
| 21 | Household owns: bed | |  |  |  |  |  |
|  |  | 0 No | 74.3 | 52.9 | 34.9 | 23.5 | 5.5 |
|  |  | 1 Yes | 25.7 | 47.1 | 65.1 | 76.5 | 94.5 |
| 22 | Household owns: electric mitad | |  |  |  |  |  |
|  |  | 0 No | 100.0 | 100.0 | 100.0 | 99.1 | 89.9 |
|  |  | 1 Yes | 0.0 | 0.0 | 0.0 | 0.9 | 10.1 |
| 23 | Household owns: kerosene / pressure lamp | |  |  |  |  |  |
|  |  | 0 No | 86.3 | 85.1 | 81.0 | 69.1 | 76.9 |
|  |  | 1 Yes | 13.7 | 14.9 | 19.0 | 30.9 | 23.1 |
| 24 | Type of cooking fuel | |  |  |  |  |  |
|  |  | 1 Electricity | 0.0 | 0.0 | 0.0 | 0.0 | 0.9 |
|  |  | 2 LPG | 0.0 | 0.0 | 0.0 | 0.0 | 0.2 |
|  |  | 5 Kerosene | 0.0 | 0.0 | 0.0 | 0.0 | 6.0 |
|  |  | 7 Charcoal | 0.0 | 0.0 | 0.0 | 0.0 | 9.5 |
|  |  | 8 Wood | 97.8 | 94.7 | 90.0 | 91.7 | 74.3 |
|  |  | 9 Straw / shrubs / grass | 0.7 | 0.8 | 1.3 | 1.8 | 3.2 |
|  |  | 11 Animal dung | 1.5 | 4.2 | 8.7 | 6.5 | 5.8 |
|  |  | 96 Other | 0.0 | 0.2 | 0.0 | 0.0 | 0.2 |
| 25 | Household has separate room used as kitchen | | |  |  |  |  |
|  |  | 0 No | 97.6 | 95.0 | 92.5 | 87.1 | 68.4 |
|  |  | 1 Yes | 2.4 | 5.0 | 7.5 | 12.9 | 31.6 |
| 26 | Animal-drawn cart | |  |  |  |  |  |
|  |  | 0 No | 100.0 | 99.6 | 99.6 | 99.3 | 97.0 |
|  |  | 1 Yes | 0.0 | 0.4 | 0.4 | 0.7 | 3.0 |
| 27 | Boat without a motor | |  |  |  |  |  |
|  |  | 0 No | 100.0 | 100.0 | 100.0 | 100.0 | 100.0 |
| 28 | Boat with a motor | |  |  |  |  |  |
|  |  | 0 No | 100.0 | 100.0 | 100.0 | 100.0 | 99.6 |
|  |  | 1 Yes | 0.0 | 0.0 | 0.0 | 0.0 | 0.4 |
| 29 | Land usable for agriculture | |  |  |  |  |  |
|  |  | 0 No | 3.7 | 9.1 | 7.2 | 9.4 | 62.1 |
|  |  | 1 Yes | 96.3 | 90.9 | 92.8 | 90.6 | 37.9 |
