# Supplementary material for: Measuring equity in utilization of emergency obstetric care at Wolisso Hospital in Oromiya, Ethiopia: a cross sectional study
Source: Int J Equity Health. 2013 Apr 22;12:27. doi: 10.1186/1475-9276-12-27 (PMC3639914; doi:10.1186/1475-9276-12-27)
Supplement: Additional file 3 — Questionnaire developed and used in Wolisso Hospital. [file 1475-9276-12-27-S3.doc]

**Supplementary files - Annex 3**

Questionnaire developed and used in Wolisso Hospital

| **1** | Name of health facility | | | | |
| --- | --- | --- | --- | --- | --- |
| **2** | Questionnaire number |  | **3** | In-patient number |  |
| **4** | Today’s date (dd/mm/yy) | ___/____/___ | **5** | Admission date (dd/mm/yy) | ___/___/____ |
| **6** | Name of your Woreda | |  | | |
| **7** | Name of your Kebele | |  | | |
| **8** | Age in completed years | |  | | |
| **9** | Parity (*after the current delivery*) | |  | | |
| **10** | What main means of transportation did you use to come to this facility? | | 1. On foot 2. Bajaj 3. Private/hired car 4. Public transport 5. Other (specify)_________________________ | | |
| **11** | How much money did you spent on transportation to come here? | |  | | |
| **12** | Have you ever attended school? | | 1. No *15* 2. Yes | | |
| **13** | If yes, what is the highest level of school you attended? | | 1. Primary 2. Secondary 3. College/university *15* | | |
| **14** | What is the highest class you completed? | |  | | |
| **15** | Highest educational attainment  (*work out from 12, 13 & 14*) | | 1. No education 2. Incomplete primary 3. Complete primary 4. Incomplete secondary 5. Complete secondary 6. Higher | | |
| **16** | Do you have a table in your house? | | 1. No 2. Yes | | |
| **17** | Does your household own a radio? | | 1. No 2. Yes | | |
| **18** | What is the main material of the roof of your main house? | | 1. Thatch/leaf/reed/bamboo 2. Wood planks/wood 3. Corrugated iron 4. Cement/concrete 5. Other (specify)_______________________ | | |
| **19** | Do you have a toilet at home? | | 1. No *21*  2. Yes | | |
| **20** | What is the type of the toilet facility? | | 1. No facility / bush / field 2. Composting toilet 3. Pit latrine without slab / open pit 4. Pit latrine with slab 5. Ventilated improved pit latrine (VIP) 6. Flush toilet | | |
| **21** | Which service did the mother receive?  (*from the mother’s file*) | | 1. Normal delivery 2. Assisted vaginal delivery 3. Caesarean section 4. Other (specify)_________________________ | | |
