# Supplementary material for: Measuring equity in utilization of emergency obstetric care at Wolisso Hospital in Oromiya, Ethiopia: a cross sectional study
Source: Int J Equity Health. 2013 Apr 22;12:27. doi: 10.1186/1475-9276-12-27 (PMC3639914; doi:10.1186/1475-9276-12-27)
Supplement: Additional file 4 — A comparison of women interviewed and those not interviewed based on selected characteristics. [file 1475-9276-12-27-S4.doc]

**Supplementary files - Annex 4**

A comparison of women interviewed and those not interviewed based on selected characteristics

| Characteristics | | N (%) | | Total | X^2^ test  P value |
| --- | --- | --- | --- | --- | --- |
|  |  | Not interviewed | Interviewed |  |  |
| District | Wolisso | 380 (50.8) | 272 (51.1) | 652 (50.9) | 0.789 |
|  | Goro | 42 (5.1) | 27 (5.1) | 69 (5.4) |  |
|  | Wonchi | 60 (8.0) | 34 (6.4) | 94 (7.3) |  |
|  | Welkite | 96 (12.8) | 73 (13.7) | 169 (13.2) |  |
|  | Becho | 44 (5.9) | 38 (7.1) | 82 (6.4) |  |
|  | Ameya | 30 (4.0) | 26 (4.9) | 56 (4.4) |  |
|  | Others | 96 (12.8) | 62 (11.7) | 158 (12.3) |  |
| Age | <20 | 82 (11.0) | 60 (11.3) | 142 (11.1) | 0.346 |
|  | 20-24 | 246 (32.9) | 195 (36.7) | 441 (34.5) |  |
|  | 25-29 | 256 (34.2) | 158 (29.7) | 414 (32.3) |  |
|  | 30-34 | 94 (12.6) | 71 (13.3) | 165 (12.9) |  |
|  | 35-39 | 52 (7.0) | 42 (7.9) | 94 (7.3) |  |
|  | 40-44 | 13 (1.7) | 5 (0.9) | 18 (1.4) |  |
|  | >44 | 5 (0.7) | 1 (0.2) | 6 (0.5) |  |
| Length of stay (days) | 0 | 100 (13.4) | 35 (6.6) | 135 (10.5) | <0.001 |
|  | 1 | 425 (56.8) | 239 (44.9) | 664 (51.9) |  |
|  | 2 | 58 (7.8) | 52 (9.8) | 110 (8.6) |  |
|  | 3 | 30 (4.0) | 24 (4.5) | 54 (4.2) |  |
|  | 4 | 20 (2.7) | 32 (6.0) | 52 (4.1) |  |
|  | 5 | 20 (2.7) | 29 (5.5) | 49 (3.8) |  |
|  | 6 | 19 (2.5) | 30 (5.6) | 49 (3.8) |  |
|  | >6 | 76 (10.2) | 91 (17.1) | 167 (13.0) |  |
| Type of delivery | Normal delivery | 668 (89.3) | 387 (72.7) | 1055 (82.4) | <0.001 |
|  | Caesarean section  or assisted vaginal* | 80 (10.7) | 145 (27.3) | 225 (17.6) |  |
| Total | | 748 (100) | 532 (100) | 1280 (100) |  |

*Forceps or vacuum extraction
